# Understanding the Political Challenge of Red and Processed Meat Reduction for Healthy and Sustainable Food Systems: A Narrative Review of the Literature

**DOI:** 10.34172/ijhpm.2020.238

**Published:** 2020-12-02

**Authors:** Katherine Sievert, Mark Lawrence, Christine Parker, Phillip Baker

**Affiliations:** ^1^School of Exercise and Nutrition Sciences, Deakin University, Geelong, VIC, Australia.; ^2^Institute for Physical Activity and Nutrition, Deakin University, Geelong, VIC, Australia.; ^3^Melbourne Law School, The University of Melbourne, Melbourne, VIC, Australia.

**Keywords:** Red Meat, Processed Meat, Political Challenge, Environmental Sustainability, Food Systems

## Abstract

**Background:** Diets high in red and processed meat (RPM) contribute substantially to environmental degradation, greenhouse gas (GHG) emissions, and the global burden of chronic disease. Recent high-profile reports from international expert bodies have called for a significant reduction in global dietary meat intake, particularly RPM, especially in high-income settings, while acknowledging the importance of animal-sourced foods to population nutrition in many lower-income countries. However, this presents a major yet under-investigated political challenge given strong cultural preferences for meat and the economic importance and power of the meat industry.

**Methods:** A theoretically-guided narrative review was undertaken. The theoretical framework used to guide the review considered the interests, ideas and institutions that constitute food systems in relation to meat reduction; and the instrumental, discursive and structural forms of power that actors deploy in relation to others within the food system.

**Results:** High production and consumption levels of RPM are promoted and sustained by a number of factors. Actors with an interest in RPM included business and industry groups, governments, intergovernmental organisations, and civil society. Asymmetries of power between these actors exist, with institutional barriers recognised in the form of government-industry dependence, trade agreement conflicts, and policy incoherence. Industry lobbying, shaping of evidence and knowledge, and highly concentrated markets are key issues. Furthermore, prevailing ideologies like carnism and neoliberalism present embedded difficulties for RPM reduction. The literature noted the power of actors to resist meat reduction efforts exists in varying forms, including the use of lobbying, shaping of evidence and knowledge, and highly concentrated markets.

**Conclusion:** There are a number of political challenges related to RPM reduction that contribute to policy inertia, and hence are likely to impede the transformation of food systems. Research on policy efforts to reduce RPM production and consumption should incorporate the role of power and political feasibility.

## Background

Unhealthy diets and poor nutrition together are the leading contributors to the global burden of disease.^1^ Diet-related chronic diseases including cardiovascular disease, cancers and type-2 diabetes are prominent causes of death and disability,^2^ and contribute substantially to current and future health-related government expenditures.^3^ Diets high in red and processed meats (RPMs) are significant contributors to this disease burden.^4^

The World Health Organization (WHO) defines ‘red meat’ as any mammalian meat (beef, veal, pork, lamb, mutton, horse, or goat), usually consumed cooked, and ‘processed meat’ as any meat that has been changed (eg, by salting, curing, fermentation, smoking) to augment flavour or improve preservation.^5^ Red meat is a source of essential macro- and micro-nutrients, such as protein, iron and vitamin B12,^6^ although with the exception of B12, these nutrients are also in plant-based foods, albeit in less bioavailable form.^7^ However, processed and red meats have also been classified as carcinogens by WHO’s International Agency for Research on Cancer (IARC).^8^ The consumption of RPM has been associated with colorectal cancer in longitudinal studies, and there is emerging evidence of associations with cancers of the stomach, pancreas, prostate, lung, and breast.^8,9^ It is not fully understood how cancer risk increases with RPM consumption, however there is speculation that carcinogens, in particular heterocyclic amines, can form during processing or cooking at high temperatures.^10,11^ Furthermore, haem iron, found in high amounts in red meat, may promote colorectal tumour growth.^12^ RPM reduction is not considered advisable or necessary in low- and middle-income countries where intake is generally low,^13,14^ and food scarcity and lack of access to food may prevent adequate nutrient intake. Although moderate amounts of red meat can be an important component of a healthy diet, dietary guidelines in most high-income countries advise limiting consumption of red meat and avoiding processed meats altogether.

RPM production also generates environmental harms. Livestock production contributes an estimated 14%-30% of all human-made greenhouse gas (GHG) emissions,^15,16^ and is the largest source of anthropogenic methane emissions. RPM production also requires a disproportionate amount of land and finite resources relative to other foods.^17^ Some forms of cattle production for example, require up to 25 kg of animal feed and 15000 L of water to produce 1kg of beef.^18,19^ As demand for RPM rises, there is increasing pressure to use intensive farming practices such as feedlots rather than pastoral grazing. This increases the demand for animal feed, mostly from intensively mono-cropped grain crops such as corn and soy, which contributes to deforestation in places like the Amazon.^21^

With the exception of pork, RPM consumption is marginally declining in many high-income countries such as Australia and the United States,^22^ and plateauing in others.^23^ Despite this declining trend, per capita RPM consumption in high-income countries is three times higher than the global average. Middle-income countries are experiencing rapid increases in per capita meat consumption,^24,25^ associated with income and population growth, increasing urbanisation, and increased international trade – characteristics of the nutrition transition.^26^ Many high-income countries play a large role in the production and export of RPM.^27^ The increasing worldwide output is estimated to lead to GHG emissions from meat production rising by as much as 80% by 2050,^28^ exacerbating the effects of climate change. The anticipated impact of climate change on human health includes increased exposure to extreme weather events, infectious diseases, poor mental health, heat-related illnesses, and food insecurity.^29^ Furthermore, intensive meat production contributes to future risks of antibiotic resistance and pandemics with zoonotic origins, such as H1N1 influenza and more recently, severe acute respiratory syndrome coronavirus 2 (SARS-CoV-2).^30^

Acknowledging the associated harms, many authoritative bodies have called for systemic meat reduction for healthy and sustainable food systems. We use the term ‘meat reduction’ to refer to *systematic efforts, involving actions throughout the food system, to reduce the production, marketing and consumption of RPM.* The Intergovernmental Panel on Climate Change (IPCC) has called for a global food systems response including major dietary change and reductions in meat production and consumption as one component in maintaining global anthropogenic warming under 2°C.^31^ A recent high-profile report by the EAT Lancet Commission on Food, Planet and Health (“The Commission”)^32^ also called for limited consumption and production of RPM as a key strategy to mitigate the negative effects of modern industrial food systems on both population health and the environment. Shifting to healthy and sustainable diets, including reducing meat consumption, would prevent an estimated 10.9 to 11.6 million deaths per year, reduce GHG emissions, help protect environmental biodiversity, and reduce competition for water and other natural resources.^32^ The Commission proposed a package of food systems policy interventions to achieve its ambitious dietary targets. These include international and governmental support to regulate trade, remove farming and agricultural subsidies for meat and feedstock, attenuate livestock production, strengthen environmental governance, and target consumer behaviour change.^32^ Effective policy action to reduce RPM would require a synergistic package of strategies that work at multiple points to address the range of actors and interests, ideas and institutions that currently support a political economy of high RPM production and consumption.^33,34^

Despite calls for action from various international authoritative bodies, there is marked disagreement on the nature of the RPM problem, the optimal degree of reduction, and the best ways to address RPM-related harms.^35,36^ Meat has been a divisive topic in the media and among policy-makers, with often conflicting interpretations regarding the associated health and environmental harms, and different views about which, if any, policy actions should be taken. Recent proposals in both the United Kingdom and Australia for a meat tax, for example, were met with strong objections in public discourse.^37,38^ Many advocate for integrated policy approaches to achieve healthy and sustainable food systems, through actions targeting multiple leverage points throughout the food system simultaneously.^35,36,39^ However, potential reduction efforts can be obstructed or limited by powerful actors in food systems, and the discursive and institutional frameworks currently shaping food policy priorities and guiding system-wide action. Meat production and processing industries in particular are major contributors to rural livelihoods and the economy of many countries,^40^ and involve a number of organised interest groups that have an interest in using their power to maintain the status quo and undermine efforts at RPM reduction.

RPM reduction presents an important, yet under-investigated, challenge to achieving the policies outlined by the Commission, IPCC and WHO. Strategies for transitioning to healthy and sustainable food systems will be vital in coming years, including actions to mitigate the harms linked with RPM. Despite research into potential approaches and policy actions for reducing RPM, such as meat taxes or consumer labelling (see Supplementary file 1 for a full outline of proposed policy actions to attenuate meat production and consumption), there is little scholarly investigation of the political feasibility of these actions in light of the contested nature of meat reduction. Acknowledging these challenges, the aim of this review is to identify and understand the key political economy challenges of reducing the production and consumption of RPM, in order to inform societal actions towards healthy and sustainable food systems.

## Methods

This study used a narrative literature review and synthesis method.^41^ This involved a scholarly summary of evidence incorporating author interpretation and critical analysis.^42^ It is appropriate for the study aim as it allows for the exploration of relationships within and between studies on a complex topic, as well thematic analysis of key findings. This method also allows for both amalgamation and analysis of a broad range of studies that fall under a variety of disciplines and formats. This is important as the topic has been investigated by a wide range of academic disciplines and organisations. The method involved four steps: (1) exposition of theory to guide both the search and the analysis; (2) a systematic search for relevant literature, with additional branching searches where necessary; (3) analysis of the literature sources and identification of key themes, and (4) thematic synthesis of the results. To strengthen the rigour of the review process, these steps were guided by the Preferred Reporting Items for Systematic reviews and Meta-Analyses (PRISMA) guidelines.^43^

###  Theoretical Framework

To guide the review, we adopted a theoretical approach grounded in political economy. This means examining ‘political, economic and social forces in society, the distribution of power and resources between different actors within and surrounding food systems, and the processes that generate, sustain and transform these relationships over time.’^39,44,45^ This approach involves understanding the power of actors to shape various elements of food systems including production inputs, supply chains, food environments and consumer behaviour, as well as the policy, regulatory and knowledge frameworks that define those systems.^46,47^ To do this, we adopted two key frameworks. First, the ‘Three I’s’ framework to consider how the Interests, Ideas, and Institutions that constitute food systems can resist or promote meat reduction. Second, was Clapp and Fuchs’ tripartite framework to understand how the instrumental, discursive, and/or structural power of actors influences these interests, ideas and institutions.^20,50,51^

####  The 3 I’s: Interests, Ideas, and Institutions 

A first step in political economy analysis is ‘actor designation’ – the identification of individuals, organisations, and groups who have an interest in meat reduction, and who deploy and accrue power within the system.^52^ Many actors have an * interest* in meat reduction, including intergovernmental organisations, governments, civil society groups, businesses, scientists and consumers. The interests of these actors in relation to meat reduction varies, including private interests (such as maintaining corporate profits), public interest (such as promoting health, reducing healthcare costs, and protecting the environment), or both (such as economic growth). These interests are often upheld and sustained by cultural variables or socially-constructed beliefs, referred to as *ideas.*^48^ Ideas refer to shared values, beliefs, assumptions and forms of knowledge about the nature of reality, that guide decision-making and behaviour.^53-55^ In the policy arena, ideas are constructed and upheld via the ‘coordinative discourses’ of multiple actors connected to an issue, including networks of experts, civil society advocates, business associations, and politicians among others, who share common interests and causal beliefs, such as whether and to what extent RPM is a problem, what causes the problem, and what solutions should be prioritised.^48,56^ Ideas that dominate within political and policy-making systems can come to define and constitute *institutions*, as the “formal and informal rules, norms, precedents, and organisational factors that structure political behaviour”^57^(p. 709, referencing Hall^48^). Institutions often, in turn, act as ideational filters by legitimising certain forms of knowledge and evidence over others.^58^ Institutions can include, for example, government policy-making processes, governance structures, laws and regulations, dominant norms within policy-making organizations or in society-at-large, and historical relationships between political decision-makers and other societal elites such as business leaders.^59,60^

####  Instrumental, Discursive, and Structural Power Framework

In order to understand the power of actors to influence the interests, ideas and institutions that constitute food systems in ways that resist or promote meat reduction, we use Clapp and Fuchs’ power framework.^20,50,51^ Where Clapp and Fuchs focus primarily on corporate power in their framework, this review will consider the power of all actors with an interest in RPM. The forms of power in this framework are described below.

*Instrumental power* is the direct influence of one actor in relation to the behaviour of another.^50^ This can include, for example, influencing policy-makers through lobbying, providing direct inputs into policy consultations, ‘revolving doors’ whereby personnel move between industry and government regulatory agencies, political donations, and financing academic activities and sponsoring favourable research.^39,50^ The food politics literature often refers to the instrumental power of ‘Big Food’ in shaping food and nutrition policies through their corporate lobbying and political financing activities in particular.^61,62^

*Discursive power *is the power to influence the underlying norms, values, and belief-systems that guide thinking and behaviour,^50,63^ as well as the apparent ‘frames’ in which issues are interpreted and openly portrayed.^64^ It can manifest in normalising ‘truths’ about a particular issue, and alter perceptions, often unconsciously, about what interpretations and solutions are considered acceptable or desirable.^39^ More obviously examples of discursive power include advertising and promotion, corporate social responsibility initiatives, and communicating scientific evidence.^65,66^ Discursive power can also underpin collective action – those who can agree on a common characterisation of a given problem (eg, that RPM is harmful to human health and the environment) may be more likely to mobilise support, counter opposition and influence decision-makers relative to those who are divided.^67^ Discursive power is also ideological – for example, the emergence of neoliberal free-market thinking and economic policies since the 1980s, has fostered strong preferences for market-based approaches to governance (eg, public-private partnerships), deregulation and a minimal role for government intervention.^68,69^

*Structural power* is the power to control the “range of choices available to others.”^50^ For example, governments often define policy agendas, may decide who participates and who is excluded from the policy-making process, and can adopt command-and-control regulation to shape the choices and behaviours of others. Corporations often adopt private standards, for example voluntary codes on responsible marketing or supply chain sustainability, with the intention of delaying or even completely replacing regulation by government.^50,63^ Globalisation has enhanced the structural power of transnational corporations, by making it easier to transfer capital investments across national borders, meaning governments must increasingly compete for those investments by creating business-friendly policy environments (eg, by relaxing regulatory standards, or providing tax concessions), over the protection of public health or the environment.^61^ Public-private or multi-stakeholder partnerships further enhance the structural power of corporations in setting public policy agendas and decision-making.^61^

###  Literature Search and Study Selection

A detailed explanation of the search process can be found in Supplementary file 2. This involved three steps. First, a scoping review was performed initially to both identify and group relevant search terms, as well as to identify an appropriate guiding framework. Consultation with a Deakin University Research Liaison Librarian was undertaken in order to ensure comprehensiveness of search terms, as well as reviewing effective search strings and suitable databases for searches. Second, four databases (Scopus, Web of Science, PubMed, and EBSCO Host) were searched for relevant studies. Search terms such as “meat,” “beef,” “livestock,” “health,” “sustainab*,” and “polic*” were used. Additional branching searches were conducted to ensure comprehensiveness of included literature, as new knowledge and references were identified. Third, the websites of authoritative organizations with a mandate or interest in improving nutrition, public health and/or environmental sustainability were also searched to identify relevant reports, policy briefs, or other documents. These included WHO, the Food and Agricultural Organization (FAO), Committee on World Food Security, IPCC, Food Climate Research Network, International Panel of Experts on Sustainable Food Systems, EAT, United Nations Standing Committee on Nutrition, International Fund for Agricultural Development, World Bank, and the World Economic Forum.

###  Analysis and Final Synthesis

All studies were uploaded to NVivo12, the qualitative analysis software.^70^ The results were then coded against the theoretical framework, and themes were identified and iteratively refined using constant comparative analysis.^71^ This allowed for nuance to emerge throughout the analytical process. The findings were then synthesised, organised in accordance with the framework in order to present the results of the analysis.

## Results

 The literature included in this review was sourced from a wide range of disciplines including public health, economics, agricultural science, food policy, and business studies. The majority of studies were from high-income countries such as the United States, the United Kingdom, Australia, and Sweden. Studies covered a diversity of policy issues and political challenges relating to meat reduction. Guided by the conceptual framework, the following sections summarise these issues and challenges.

 First, we discuss how high levels of RPM production and consumption are sustained through interests, ideas and institutions that have historically constituted food systems, starting with an introduction to the relevant actors and their interests. Second, we then review the instrumental, discursive, and structural power of actors.

###  Actors and Their Interests

 Actors identified in the literature with an interest in meat and meat reduction include governments, civil society groups, businesses and industry groups, researchers and consumers. The majority of the literature focused on the power of the meat industry in relation to meat reduction, however other key actors were also acknowledged including novel protein industries (such as plant-based imitation meat or cell-cultured meat), governments and international organisations, and civil society groups and citizens.

####  Businesses and Industry Groups

The literature identified the meat industry as the most powerful interest group promoting high levels of RPM consumption, and resisting reduction efforts. The term ‘meat industry’ refers to ‘market’ actors specifically – those that have private interests, are for-profit and comprise the modern industrial livestock sector, including production (including animal feed and pesticides), packing, preservation, retailers (such as supermarkets and fast food chains), representative peak organisations, as well as ancillary service providers such as the advertising industry. The most powerful RPM corporations identified in the literature were JBS (Brazil), Tyson Foods (USA), and WH Group/Smithfield (China). These three corporations dominate the global market. At the time of writing, they account for 63% of global market share for pork; with Tyson and JBS controlling 46% of the market for beef.^72^ Animal feed and technology input suppliers such as Cargill (USA) and Bunge (USA) were also noted to be powerful corporate players, given their sizeable reach as some of the largest agricultural commodity traders in the world.^40^ Together, these can be collectively referred to as ‘Big Meat.’ In addition to these global, large-scale, industrial companies, the meat industry also consists of small- and medium-sized players in domestic markets, including farmers and local industry associations. The market structure varies by country in this regard, for example, the US meat market is highly concentrated with large global corporations^73^ whereas in countries like New Zealand, the market is characterised by a diversity of domestic producers.^74^

A small number of studies identified alternative protein industries as actors who stand to gain from RPM reduction. Alternative proteins encompass four primary categories – traditional vegetarian proteins, novel plant-based proteins, edible insects, and ‘cellular agriculture’ (lab-grown meat).^75,76^ Some corporations participate in both the alternative protein and RPM markets. For example, many fast food retailers are diversifying into plant-based meats as a means to expand their marketing options and to be seen as market leaders ‘shifting (with) the agenda.’ As the call for reduced meat consumption has gained traction in the public sphere, replacing meat with these alternatives has become a more financially lucrative opportunity. This has also caught the attention of Big Meat and powerful animal feed corporations, leading many to acquire alternative protein start-ups and position themselves as market leaders in the area. For example, Cargill increased investments in 2019 into Puris, a pea protein production company, of up to $75 million.^77^ Other animal feed companies are following suit. Bunge Ltd, one of the largest soy and grain traders globally, purchased a 1.6% stake in Beyond Meat in 2019, showing remarkable foresight as the market capitalisation of Beyond Meat was around $9.9 billion at the time of writing – significantly greater than Bunge Ltd, despite being a firm with over 30000 employees and existing in the market for over 200 years.^78^

####  Governments and Inter-governmental Organisations

In many countries, the RPM sector is economically important to governments given its contribution to gross domestic product, tax revenues, and exports and employment, especially in rural areas. In Australia, for example, the economy is reliant on the industry as one of the key exporters and a major source of employment, particularly in rural areas where over 191000 people are directly employed by the meat and livestock sector.^79^ Furthermore, a variety of government departments have a mandate that includes supporting the RPM sector alongside other roles and responsibilities, including agriculture, health, food standards and the environment, creating a challenge of policy coherence and often leading to conflicting objectives.^80^ For example, a department of agriculture in a given country may be responsible for policies supporting the RPM sector (such as farming subsidies) and for environmental protection (such as water usage).

Unlike commercial actors where profit is the predominant driving force behind decision-making, governments can have a widespread agenda with multiple interests at play, including the health of their citizens and environmental concerns. For example, China’s government has included a recommendation to reduce RPM consumption to 40-75 g of meat per day in their dietary guidelines.^81^ These recommendations were distributed to citizens through a series of public information advertisements, with environmental conservation at the core of the message. It is estimated that almost one billion tonnes of carbon and methane emissions from the livestock industry could be reduced from China’s emissions output should these guidelines be widely adopted.^81^

Some studies recognised the role of intergovernmental organisations as both facilitators and barriers to RPM reduction. Intergovernmental organisations such as WHO, FAO, IPCC, and the High Level Panel of Experts on Food Security and Nutrition (HLPE) play largely technical, normative and convening roles in global food systems governance. This includes, for example, producing policy reports and guidelines such as the highly-cited “Livestock’s Long Shadow” by FAO,^82^ “Climate Change and Land” by IPCC^83^ and “Sustainable agricultural development for food security and nutrition: what roles for livestock?” by HLPE.^84^ Technical work, for example the WHO-International Agency for Research on Cancer (IARC) “Monographs on the evaluation of carcinogenic risks to humans: red meat and processed meat”^8^ help to provide an evidence base for informing meat reduction.

The Codex Alimentarius Commission (Codex), the United Nations body administered under the joint FAO/WHO Food Standards Programme, has a dual mandate to protect public health and safety while facilitating international food standards harmonisation and trade. Codex standards are important because they inform food-standard setting by governments. Interestingly, Codex ‘commodity-specific’ committees relevant to meat including the Committee on Meat and the Committee on Processed Meat and Poultry Products were abolished in 1973 and 1990 respectively, and the Committee on Meat Hygiene adjourned indefinitely in 2005.^85^ However, meat products fall under General Subject Committees, for example those mandated to set standards on additives, labelling and hygiene. Codex standards are also important as reference standards in trade agreements, including in the World Trade Organization’s (WTO’s) Technical Barriers to Trade and Sanitary and Phytosanitary Measures Agreements.

Trade policy and/or conflicts with WTO rules were a barrier cited in the literature for some member states attempting to regulate the import and export of RPM.^23,32,86-98^ For example, in order for Samoa to join the WTO, the government had to reverse an already-implemented restriction on fatty meat imports that had been employed as an anti-obesity measure.^23^ In another example, the European Community (EC) banned the import of meat products containing artificial hormones, leading to a sizeable dispute between the EC and the United States in the WTO Dispute Settlement Body as a result.^99^ The WTO ultimately ruled against the EC. In contrast, the development of national standards in Ghana led to the restriction of imports of some high-fat meats and reduced the availability of these products in the Ghanaian food supply, whilst still adhering to global trade law.^100^

####  Civil Society Groups and Citizens 

The literature cites a number of civil society actors with an interest in meat reduction. Civil society actors can be defined as “public interest non-governmental organisations, social movements, research organisations and academics, communities and consumers.”^39^ For example, in the United States, the Reducetarian Foundation, works to form networks between environmental, health, and social justice organisations and individuals to promote RPM reduction through public education and engagement.^101^ The collective power of these groups can also pressure governments to take action. This is evident in Denmark where a “meat tax” was proposed to Parliament following the release of a publication by the Danish Council of Ethics, an influential think-tank.^102^ Whilst the tax did not eventuate in law, it demonstrates the important norm-promotion role that civil society groups can play. Furthermore, public education surrounding the issue of RPM and its associated harms has markedly increased in part due to signature reports from research groups such as International Panel of Experts on Sustainable Food Systems,^40^ EAT-Foundation,^32^ World Cancer Research Fund,^9^ and the World Wildlife Fund.^103^

Some civil society groups have been reluctant to advocate for significant reductions in RPM consumption. One study noted that strategic considerations and feasibility of the message (ie, not wanting to disconnect from the mainstream views about meat) was a primary reason for this.^104^ Furthermore, depending on the motivation of the varying groups, efforts to influence policy can be stymied as a result of inconsistent messages about the amount of meat reduction needed versus advocacy for the complete removal of RPM consumption. For example, an environmentally or public-health motivated advocate may be content with simply reducing RPM consumption,^104,105^ however an animal advocate would argue that only complete removal of RPM can meet their goals.^106^ Within this, the extent of reduction can vary depending on the outcome of human health or carbon emissions.

A small number of studies also noted the role of vegetarian movements endorsed by civil society groups. For example, in the United States, ‘Meatless Monday’ is a public health initiative revived by Johns Hopkins School of Public Health in 2003 (having originated in World War I) across the country.^107^ The campaign aimed to reduce people’s meat consumption incidentally by encouraging schools, hospitals, dining services and individuals to abstain from meat one day a week.^108^ Local organisations such as Compassion Over Killing, a not-for-profit in Los Angeles, were strong advocates of the movement.^109^ This campaign, in addition to other promotions of plant-based meals such as recipe cards,^110^ represents a strong opportunity to normalise diets that are less meat-heavy.

###  Ideas

 Literature about ideas that formed barriers or enablers of RPM reduction policy fell under three broad themes – carnism, the production and communication of knowledge and evidence, and contestation of optimal policy solutions.

####  The Ideology of Carnism 

Deeply entrenched in most cultures is the idea that ‘a meal is not a meal without meat.’ In some studies, this ideology is referred to as ‘carnism,’ first coined by sociologist Melanie Joy.^111^ Carnism contends that the choice to eat meat is an ideology that positions consumption as “natural, normal, and necessary”^111^ on the basis that humans evolved to eat meat, and survival and strength depend on it.^112,113^ Preferences for red meat in particular have colonial origins, where Europeans were depicted as strong and superior in contrast to the ‘weak’ and ‘feminine’ First Peoples of their colonised nations, whom consumed largely plant-based diets.^114^ Norms and narratives around eating meat have been cited as a significant reason behind the policy inertia in this area.^97,115-128^ Policies aimed at reducing RPM consumption – in high-income countries in particular – would require a “profound societal transition” as the value of meat in these contexts is high, and often one of the more popular food products in many countries.^116^ Carnism and consumer demand reinforce each other in a causative loop, as demand levels have pushed meat production to intensive levels, allowing meat prices to fall, thereby helping to sustain high levels of consumption.

Meat is also deeply tied to social identity, in particular masculinity, as eating red meat has become synonymous with increased strength and “being a ‘real’ man”^129^ The association of meat with masculinity has contributed to higher levels of meat consumption among men compared with women in the United Kingdom, the United States, and other high-income populations.^130,131^ Carnism may also underlie attempts to reserve particular terminology only for RPM. In the US state of Mississippi, legislation passed stipulating that plant-based food products were prohibited from being labelled as “meat” or “meat food product” (for example, ‘vegan bacon’ or ‘veggie burger’), following intense lobbying from meat industry lobby groups,^132,133^ concerned that the ideological threat of these imitation products may impact ‘traditional’ meat profits. In July 2019, the Plant Based Food Association and member company ‘Upton’s Naturals’ filed a lawsuit against the legislation,^133^ however this was subsequently dropped.^134^ This is an example of ‘discursive power’ as corporations seek to retain RPM as “real” and “natural” in comparison to plant-based counterparts.

One study acknowledged that cognitive dissonance leads meat consumers to avoid or resist evidence of the negative consequences of meat eating.^135^ Social and cultural norms in this regard have been described as powerful and pervasive,^135^ including the dissociation of meat from its animal origin in the language used to describe it (eg, using “beef” instead of cow, “pork” instead of pig).^136^ A recently published qualitative study from Australia describes persistent underestimation of the environmental impacts of red meat by consumers.^137^ Considerably less public scrutiny has been placed on RPM industries than the fossil fuel industry, despite the relatively similar GHG emissions output. The Institute for Agriculture and Trade Policy shows that combined, the top five meat and dairy companies globally (JBS, Tyson Foods Inc, Cargill, Dairy Farmers of America and Fonterra Group) emit more GHGs than large-scale oil corporations such as Exxon Mobil or Shell.^138^ Consumers can also be apathetic to the environmental risks of RPM. In a survey of supermarket shoppers conducted by Meat and Livestock Australia in 2019, the main consumer priorities were price, quality and freshness, with no mention of animal welfare or environmental concerns.^139^ This policy omission links with the ideological premise that meat is a normal and necessary part of daily life, and responsibility for GHG emission reduction should lie with other ‘less natural’ industries, such as plastic manufacturing or fossil fuels.

####  Neoliberalism and Productivism

Another key theme was how high levels of meat production and consumption are enabled by deeper belief systems that preference free markets and economic growth over health and environmental objectives. Many countries have embraced neoliberal ideologies that promote ‘free-market’ economic policies, deregulation and a minimal role for government intervention. This has allowed for the substantial growth in size of transnational food corporations, with power shifting away from both state and civil actors and governance being positioned in a more market-orientated form.^39^ This growth has only been amplified with the financialisation of much of the global food economy, involving the growth in marketised securities, monetary exchange freedoms, and financial sector deregulation.^140,141^ Neoliberalism reinforces, and is reinforced by a ‘productivist’ policy paradigm, which has formed the basis for agricultural law and policy, especially in high-income countries, since the 1960s. This manifests in the promotion of agricultural efficiency, high crop-yields, export-orientated growth, and technology and resource-intensive inputs.^142^Policy outcomes linked with productivism include widespread use of intensive factory farming and the overproduction of meat, particularly in high-income countries.^143,144^ Lang et al note that while this has allowed for lower priced meat for consumers, it encompasses hidden, or unaccounted for, costs, such as the cost of climate change or healthcare costs associated with antibiotic resistance.^123,142^

###  Institutions

Institutions are the formal and informal ‘rules of the game’ that actors follow as they endeavour to achieve their goals and interests.^59^ They can include formal structures like historical policy priorities or private-public partnerships, but also can include informal arrangements such as norms that guide policy-making. Major themes that emerged from the literature include financial relationships between governments and RPM industries, trade agreements and investment law, and policy incoherence.

####  Institutionalisation of Government-Meat Industry Co-dependence

Given the sizeable economic impact of RPM and its cultural importance within many societies, the stability of the meat industry is also a key interest for many governments. In Australia, the red meat industry generates returns of above $22 billion annually.^79^ This is in part thanks to the Australian government’s commitment to provide the agricultural sector with “research and development funding, levy monies and facilitation of the management of issues of national importance.”^145^ Government subsidies in a number of countries have historically been employed as an important function of ‘productivism.’^142^ Over time these subsidies have led to a surplus of foods and a multitude of human and planetary health problems. The Food and Land Use Coalition estimated that of the over US$700 billion given annually across subsidy programs worldwide, around $530 billion is paid to agricultural farmers.^146^ In the United States, 63% of subsidies are directed towards meat and dairy.^147^

As a result, many studies identified a co-dependency between governments and the RPM industry, being perpetually reinforced. One study found that any efforts by policy-makers to reduce RPM consumption would likely result in the mobilisation of “powerful interest groups.”^135^ In the United States, for example, animal agribusinesses are worth around $125 billion, and are highly concentrated. This market concentration is supported by government subsidies which generally favour dominant firms over smaller competitors.^72^ For example, as the largest RPM producer in the United States and the second largest in the world, Tyson Foods Inc. receives discounted corn and soybeans for animal feed and direct payments to farmers from the US Department of Agriculture (enabling savings of $288 million USD per year).^72^ Attempts by governments to regulate or enforce technological reform on the industry have therefore been evaded due to the weight of “well-connected, large-scale commercial productions.”^82^ Furthermore, many politicians in countries like Australia see supporting the red meat industry as integral to retaining electoral seats in swing states,^80^ and thus are reticent to challenge the status-quo.

####  Trade Agreements and International Investment Law

The growing number of regional and bilateral trade agreements, in addition to the wider structures of international trade and investment law (including investor-state dispute settlement mechanisms and arbitration bodies), have contributed to significant challenges for domestic policy-making in relation to meat reduction. To date, food safety and livestock disease are the primary considerations given for restricting imports of meat products, rather than health and environmental concerns.^23^ Countries compromising on existing public health policies in order to join the WTO and other regional and bilateral trade and investment agreements (as exemplified earlier with Samoa). These policy conflicts can continue after countries accede to the WTO, as member states can engage in trade policy review processes and arbitration to challenge the regulations adopted by other governments. The threat of trade arbitration can also result in ‘regulatory chill,’ whereby policy-makers are deterred from taking regulatory action in the first place.^148^ Furthermore, the proliferation of preferential trade agreements that go beyond the WTO in terms of depth and scope of provisions are also key considerations. For example, the lowering of trade barriers between the US and Mexico under the North American Free Trade Agreement, saw a significant increase in meat products being imported into Mexico between 1994 and 2008.^149,150^

####  Policy Incoherence

The literature observed a diversity of approaches to meat reduction, highlighting a difference between siloed or targeted approaches compared with systemic ones involving coherent policies and actions across multiple sectors. Studies tended to demonstrate a preference for isolated or one-off regulatory proposals, usually aimed only at either production or consumption. Meat taxes (either point-of-sale or production-based),^86,87^ consumer labels,^151,152^ or innovations in agricultural technologies^118,153^ were the most commonly promoted policy proposals but rarely synergistically, and for the most part burdening the consumer. This highlights that existing governance structures in most countries are strongly institutionalised, with a tendency towards siloed approaches, subsequently resisting the adoption of system-wide packages of policy actions. However, as mentioned above, expert bodies such as The Commission^32^ have stipulated that transforming the food system – including reducing RPM production and consumption – will require a range of policy actions that work synergistically across multiple food systems drivers. Despite this, a substantial number of studies proposed ‘silver-bullet style’ or individual policy actions for RPM reduction, not packages of actions that addressed multiple drivers. Implementing siloed policy actions as a means of addressing the ecological harms associated with RPM production neglects to address the interconnected challenges of the wider system,^154,155^ however this has historically been the process for policy development due to the pre-existing nature of policy-making.

###  Instrumental Power

The literature generally identified the meat industry as the actors with the most instrumental power. A broad range of literature examines the power of large transnational companies with a disproportionate level of power in the food system. Examining the use of power by these companies in the food system, ie, “Big Meat,” is central to understanding why policies do and do not get made. The term “Big Food” has been applied by researchers and civil society to describe large-scale transnational food and beverage corporations such as Nestle and Coca Cola, actors that are incredibly powerful in the food system and actively utilise their power to maintain favourable market environments, in many cases prioritised over health and/or environmental impacts.^61,156,157^ Many of the strategies and forms of power used by these industries are being replicated by the meat industry in response to any suggestion of policies designed to reduce RPM.^80^

Given that in many high-income countries the livestock sector constitutes around 40% of total agricultural output,^158^ and processing and retailing also constitute substantial economic sectors in domestic markets around the world, these actors can apply varying forms of instrumental power when it comes to influencing regulation or policy.^51,116^ For example, many studies reveal lobbying as a key practice in circumventing policy targeted at meat reduction. In 2018, Tyson Foods spent US$1.1 million on US federal lobbying alone,^159^ dominating the lobbying spending efforts of other meat processing corporations and resulting in it being one of the most powerful companies domestically. In both the United States and Australia, meat industries applied considerable pressure throughout the development of their respective national dietary guidelines, later was acknowledged by various organisations to be a strong influence on the final recommendations that omitted any mention of reducing meat consumption^23^ or “environmental sustainability” considerations.^160^ Peak representative organisations also engage on behalf of these companies, such as the National Pork Producers Council in the United States, which invested over US$2.4 million lobbying against proposed legislation aimed at addressing water quality, antibiotic use and trade in the meat sector.^161^ Even in the context of the global SARS-Cov-22019 pandemic, Tyson Foods lobbied the Trump Administration to reopen processing factories despite slaughterhouses being considered “coronavirus hot spots” and a clear risk to workers.^162^ Furthermore, related input corporations such as Cargill, and those involved with distribution (such as food retailers and restaurants) have also engaged in extensive lobbying activities.^163^ In Brazil this has been done in order to privatise land, that more often than not has belonged to indigenous or intergenerational farmers, in order to sell to transnational corporations or foreign countries, is also commonplace and an example of instrumental power.^51,164^

###  Discursive Power 

####  Framing the RPM Reduction Problem and Solutions

Discursive power is evident in the frames used by actors to interpret and portray the RPM reduction problem and what solutions are considered optimal or desirable. There is much contention over whether or not RPM harms population and/or planetary health. Various studies have demonstrated that despite the mounting evidence to the contrary, media coverage and consumer consciousness of the problems of meat tend to be low.^165,166^ Furthermore, meat reduction has frequently been framed as extremist, associated with the ‘vegan agenda,’ by many individuals and interest groups, including the meat industry.^167,168^

As described earlier, the Commission proposes a package of food systems policy interventions to achieve its ambitious dietary targets. These include international and governmental support to regulate trade, removing farming and agricultural subsidies, attenuating livestock production, strengthening governance around environmental resources, and targeting consumer behaviour change.^32^

However, some proposals are promoted more than others, connecting to wider societal values of technological innovation and ideas of personal responsibility. For example, agricultural technologies that use less water and improve manure and herd management, nitrogen efficiency, and feeding practices, as a means of reducing emissions associated with RPM production are heavily favoured by industry groups.^118,169^ Ruminant meat production is in a unique position in this regard, as not only are there opportunities to reduce carbon emissions from the farming process, but also to facilitate carbon sequestration ie, pulling and absorbing carbon from the atmosphere. This is because both grazing and excretions from ruminant animals can stimulate plant growth and carbon fixation, making nitrogen in particular more available to the next generation of plants.^23,121^ Solutions like these shift blame away over their production practices, whilst simultaneously positioning themselves as ‘part of the solution’ – a commonly utilised mechanism of influence by other industries such as “Big Soda.”^61,170^ However, there is little consensus around whether these ‘negative emissions technologies’ are sufficient to meet emission reduction goals needed for safe planetary boundaries,^31,171^ and especially not as an isolated measure.^172^

A number of studies supported by industry or industry interest groups (such as Meat and Livestock Australia) propose consumer labelling as a policy action.^33,34^ Sonoda et al found that three key consumer values influenced the effectiveness of labelling for meat; (*i*) openness to change, (*ii*) self-enhancement, and (*iii*) security.^173^ However, these values – and thus the effectiveness of consumer labels – have been noted to be superseded by price and taste preference by Van Loo et al.^174^ It is unsurprising that industry would advocate for labelling schemes given the onus of this style of policy action lays in the hands of consumers and directing consumer choice, rather than governing land use or applying limits to RPM production practices, which would require major structural and financial reform for the RPM sector.^175,176^ Policy solutions such as labelling also reinforce ideas of ‘personal responsibility’ and put the onus onto the consumer to make the changes required to mitigate the effects on sustainability and health.^177^

Many academics have acknowledged the limitations of such siloed approaches to policy-making and have reinforced the need for systemic and multi-issue responses. Parker and Haines argue that regulation needs to adapt to make ecology a primary concern (known as ‘ecological regulation’), stating that human governance systems need to operate within ecological limits for humans, whilst ensuring social and economic pressures are included as part of a wider synergistic approach.^154^ This type of regulation might include measures such as penalising atmospheric emissions by high-contributing industries, such as the fossil fuel or agricultural sectors.^154,155^ Others advocating for integrated responses espouse the importance of incorporating sustainability across multiple policy areas, including that of dietary guidelines.^36,178^ Furthermore, the unequal balance of power among various actors in the food system was noted in the literature, suggesting that stronger state-led and participatory models of governance will be required to achieve RPM reduction.^36,39^

####  Producing and Influencing Knowledge and Evidence

The use of knowledge and evidence, as well as the power to produce, shape, disseminate and contest ideas, and narratives within these, is an example of discursive power. Establishing a sound body of evidence to support meat reduction is an important prerequisite for policy development and gaining public support. This power can be utilised by public health academics through, for example, the publication of evidence that elucidates the risk of colorectal cancer from excessive RPM consumption, which contributes to the idea that there are health harms associated with RPM, and therefore creates an idea that consumption should be reduced (red meat) or avoided (processed meat). Several major systematic reviews support this, including the WHO IARC review.^179^

RPM industries and their representatives have sponsored research or funded academics as a means of discrediting, or at the very least, disputing some of these concerns. For example, a 2011 study funded by the US National Cattlemen’s Beef Association^180^ found no correlation between RPM consumption and colorectal cancer. Similarly livestock industry associations such as Meat and Livestock Australia have provided ongoing financial support to the Australian Commonwealth Scientific and Industrial Research Organisation, leading to allegations of biased outcomes.^181^RPM industry support has also been provided for research published in international publications, such as the widely publicised review of RPM on health outcomes in October 2019 in *Annals of Internal Medicine*. The study authors had received funding from Texas AgriLife Research, as well as the International Life Science Institute, an industry front group.^182^ The study concluded that the quality of evidence for RPM-related health harms was low.^183^ It was accompanied by a clinical guideline recommendation for adults to “continue current processed and unprocessed red meat consumption” due to the low-certainty evidence.^184^ The disputing of evidence about the impacts of RPM make policy development processes more difficult as they are used by industry to refute regulatory attempts.^185^

###  Structural Power 

####  Concentration Within Global and Domestic Meat Markets

A recurring theme in the literature was the structural power connected to highly concentrated global and domestic meat markets. Highly concentrated markets give companies considerable power to reduce their own costs by imposing price restraints on suppliers and at the same time setting private standards for product processes and quality.^40^ This forces meat suppliers to become more efficient, which drives market concentration across the meat supply chain. It also enables companies to provide cheaper meat products to consumers, further perpetuating meat consumption.^35,186^ Market power also enables companies to leverage power over consumers, by constraining the choices available in markets.^50,51^ Although many meat markets are primarily domestic, there are several highly-capitalised multinational corporations that operate across dozens of countries, engaging in trade and competing with medium- and small-sized local businesses. Globally, four firms (Tyson Food – 24%, JBS – 22%, Cargill – 19% and National Beef – 10%) control 75% of all beef-processing and four firms control 70% of pork processing (WH Smithfield – 26%, JBS – 19%, Tyson Food – 17%, Orwell – 8%).^40^ In recent decades these corporations have accrued considerable market power by concentrating vertically and horizontally across and within supply chains through mergers, acquisitions and other anti-competitive market activities. The structural power of these companies can lead to the leveraging of capital mobility in order to achieve policy concessions.^39^

Nowhere has a government been more overt in their protection of RPM than in Brazil. Since 2018, the Bolsonaro administration has actively removed barriers to deforestation of the Amazon in favour of livestock land clearance.^187^ This has included budget cuts of 95% to the National Policy on Climate Change, introducing bills to reduce environmental requirements, and attempting to decrease the authority of the National Indian Foundation, which protects indigenous land rights.^164^ This is a clear example of structural power where concessions are made to domestic and transnational companies to attract investment as well as the jobs and revenue they provide, even if much of the benefit ends up offshore.

####  Government and Public Procurement

The literature noted some capacities for governments to play in support of meat reduction. Whilst partnerships between government and civil society are not widely discoursed in this area, there are a few noted opportunities for cooperative-style regulation for meat reduction. Procurement policies (such as meals in schools, hospitals, and catering in government departments) present openings for institutionalised reduction of RPM.^160,163,188^ Governments and civil society can use procurement policies to challenge the power of industry by being role models as well as reducing demand for RPM production and consumption.^151^ This strategy has been used in many European countries to increase sales of organic food and increase the acceptability and preferences of organic food to the consumers through hospital cafeterias and schools.^151^However, health advice for procurement policies are often informed by national dietary guidelines, which can be heavily influenced by industry.^163^

## Discussion

 Reducing the production and consumption of RPM is a key area of reform needed to meet global environmental and health targets, such as limiting anthropogenic global warming to no more than 2°C and attenuating global levels of chronic disease. However, the means by which this is to be achieved is contested, and political factors that may enable or constrain the implementation of meat reduction policies is under-investigated. The aim of this literature review was to identify and understand key political economy challenges of reducing the production and consumption of RPM, in order to inform societal actions towards healthy and sustainable food systems.

How these political factors may enable or constrain the implementation of policy actions were summarised against a conceptual framework incorporating interests, ideas, and institutions as categories,^48,49^ and a power framework including instrumental, discursive, and structural forms of power.^20,50,51^ This review identified the varying political factors that stand to either enable or constrain the systematic efforts to reduce the harms associated with RPM production, marketing and consumption.

 The empirical findings reviewed demonstrate the intricacy of the RPM problem and why policy aimed at addressing this problem has remained largely stagnant. It highlights the need to address actors, ideas, and institutions; and the ways in which power is currently supporting the production and consumption of RPM in the food system. A consistent pattern was observed across the potential barriers and/or enablers for RPM reduction. Firstly, most barriers to RPM reduction identified in the literature involve industry actors and commercial interests, including trade, market concentration, and for some countries, government-industry dependence. This is not surprising given that RPM, like other foods, are generally regarded as commodities to be traded for profit. Broader ideas and ideologies, including carnism and neoliberalism, also protect industry interests. A paradigm shift in mainstream policy-making would therefore be a vital component in addressing the barriers to RPM reduction.

This review did identify enabling actors who may assist in furthering policy efforts around RPM reduction, including governments, inter-governmental organisations such as WHO, FAO, and the IPCC, and certain non-governmental organisations. Currently these actors wield less instrumental and structural power than that of industry and industry interest groups, and subsequently are prevented from actioning effective change within the food system. For example, government and meat industry interests are inextricably linked in relation to jobs and economic benefits. Addressing this power asymmetry should be a core component of the food system transformation, and could include realigning incentives in the food system to reduce market concentration, restructuring governance structures that provide more widespread corporate oversight, and building more equitable supply chains under a new economic paradigm (and shifting away from productivism).^40^ Further opportunities exist for civil society groups to work in cooperation with government to gain power over industry and promote meat reduction, however this is likely to also require a shift in ideology and paradigms – most notably, the ideologies of carnism and productivism. The discursive power of consumer concern for their own health, and for some, the environment, may be a possible resource for change.

In contrast to Parker and colleagues’ call for holistic, ecologically-focused regulation,^154^ policy proposals to attenuate RPM production and consumption have been suggested primarily in isolation and not reflected within a food systems lens (for example, several studies modelled effects of a ‘meat tax’ as a sole policy proposal). Furthermore, the political viability of implementing these suggested policy actions has largely not been considered in the studies. This is understandable, as political economy of health outcomes and issues within the food system is an emerging area of research.^189^While public health nutritionists have focused on the political economy of ultra-processed foods and sugar sweetened beverages, little attention has been paid to the political economy of meat. Although a range of other disciplines do actively consider RPM production and consumption (for example geographers and business researchers), a political economy lens may be outside the scope of their usual research approach. This opens up prospects for further research that is focused around political feasibility of meat production proposals and opportunities for systemic change.

Whilst the complexity and nature of the RPM problem may make it seem that policy efforts aimed at reduction are a fool’s errand, previous instances of ‘wicked problems’ have been successfully overcome. Tobacco was seen as a largely insurmountable public health issue in many countries for decades.^190^ However comprehensive and multi-sector strategies have constrained the transnational power of tobacco companies like Phillip Morris (instrumental power), emphasised the health harms associated with smoking (discursive power), and underscored the importance of policy networks (structural power).^80^ As a result tobacco control has, for the most part, been counted as a significant public health success. The prevailing feature of success in that instance was not a sole policy action, but rather, many, at multiple levels and areas of governance.^80^

###  Strengths and Limitations

 This is the first study to conduct a critical political economy review of evidence around RPM reduction, incorporating the 3 I’s framework with Clapp and Fuchs’ power framework. It also adopted a multi-disciplinary approach, through a search across three databases in order to capture the widest array of literature possible.

 This review has several limitations. Firstly, our search strategy may have omitted relevant studies due to the size of scope. This omission could have affected the number of studies included in the analysis due to a number of literatures being inadvertently excluded. Furthermore, as is consistent with narrative review methods, the results of this study are inherently subject to bias and cultural interpretations of the authors. Determining and integrating complex interactions between themes and ideas is difficult, particularly with large sets of studies.

## Conclusion

 Achieving healthy and sustainable food systems in the wake of urgent calls to address their contributions to poor human health and environmental degradation should be multifaceted and address multiple levels of the food system. However, understanding the role of power in working towards this transformation has been critically under investigated. At the root of any human system is power, and the food system is no exception. In the context of RPM, high consumption levels are an outcome of widely prevailing asymmetries of power that have gone largely unchallenged. Therefore, research around policy efforts to reduce RPM production and consumption cannot omit the role of power.

 This area of research is dynamic and has a rapidly moving and changing set of agendas, stakeholders, science, and politics. New issues are likely to arise and politics will shift over the coming years and decades. However, the negative impacts of continued RPM production and consumption on the environment, loss of natural resources, and the rising temperatures of our planet, will ultimately affect everyone, suggesting that sooner or later enough actors will realise it is in their interests to act together to reduce RPM production and consumption.

## Ethical issues

 Not applicable.

## Competing interests

 Authors declare that they have no competing interests.

## Authors’ contributions

 KS and PB conceived the original topic for investigation and its aims. KS and PB designed the study, with contributions from ML and CP. KS was responsible for the literature search, analysis, and drafting of the manuscript. KS, PB, CP, and ML provided a critical review of the manuscript. All authors contributed to and agreed on the final version.

## Authors’ affiliations

^1^School of Exercise and Nutrition Sciences, Deakin University, Geelong, VIC, Australia. ^2^Institute for Physical Activity and Nutrition, Deakin University, Geelong, VIC, Australia. ^3^Melbourne Law School, The University of Melbourne, Melbourne, VIC, Australia.

## Funding

 Katherine Sievert was supported by a National Health and Medical Research Council (NHMRC) Postgraduate Award scholarship.

## Supplementary files


Supplementary file 1 contains Table S1.^23,32,87-98,105,115-118,120-125,127,151-153,163,173,188,190-240^

Supplementary file 2 contains the detailed explanation of the search process.^41-43,70,241,242^
