# Supplementary material for: Understanding the Political Challenge of Red and Processed Meat Reduction for Healthy and Sustainable Food Systems: A Narrative Review of the Literature
Source: Int J Health Policy Manag. 2020 Dec 2;10(12):793–808. doi: 10.34172/ijhpm.2020.238 (PMC9309962; doi:10.34172/ijhpm.2020.238)
Supplement: Supplementary file 1 — contains Table S1. 23,32,87-98,105,115-118,120-125,127,151-153,163,173,188,190-240 [file ijhpm-10-793-s001.pdf]

Supplementary file 1

Table S1. Policy Actions (Proposed and Existing) Aimed at Reducing Consumption and/or Production of Red/Processed Meat

**Policy action domain**		**Policy action example**
Governance (including policy and regulatory frameworks)	Dietary guidelines reform	*Holistic dietary guidelines* - reform national dietary guidelines to consider both health and sustainability, including the promotion of reduced/minimal RPM consumption [153,188,191-193]
	Governance structures	*Corporate Social Responsibility* – Self-regulating business model that promotes companies to be environmentally accountable [194]
Food supply	Fiscal measures	*Investment in agricultural technologies* – Government investment in technologies that assist farm production to be more environmentally efficient, eg, water management systems, more efficient carbon sequestration [92,118,121,153,193,195-215]
		Carbon/emissions tax – eg, production taxes on an agricultural level around GHG emissions (incl. nitrogen) [23,32,87,88,91,93,94,117,120,122,151,153,191-193,206,212,216-222]
		Environmental practice incentive/subsidy – Subsidise or give financial/other incentives to farmers who adopt environmentally friendly methods of RPM production [32,87,88,116,123,153,198,206,207]
		Agricultural subsidies (removal of) – Remove current subsidies that promote intensive animal agriculture [97,125,195,201,216]
		Production quota – set limits for animal agriculture production [151,201]
		Ecological compensation/true pricing - Integrating health and environmental externalities into food pricing and/or government/commercial accounting methods [115,218]
		Biodiversity offsets – any predicted biodiversity impacts in agricultural practices must be minimised and/or reversed, and then remaining impacts should be compensated elsewhere [115]
		Regulation of land clearing – Implement limits on the amount of land able to be converted for agricultural purposes [125]
	Waste management	*Reduction of food waste* – No specific definition was mentioned in the studies, however an example of a policy action would be adding value to non-utilised fruits/vegetables that ordinarily would not meet market acceptability [118,121,124,214]
	Trade	*Import tax* – Adoption of tariffs on imported RPM products [95,96,163,223]

		Trade policy –Various options were suggested in the literature, for example, removing free trade of food commodities that generate high greenhouse gas emissions i.e. red meat [96,198]
		Tradeable quotas (eg, nitrogen) – implement set limits for trade of high nitrogen producing food commodities i.e. red meat [127,151]
		Regulation of international corporate tax havens – Increasing transparency of off-shore tax processes in low-regulation countries such as Bermuda, in order to remove anonymity of investments made in harmful sectors [224]
Food environment	Fiscal measures	*Meat tax* - consumption tax designed to increase the cost of red/processed meats[32,89-91,97,98,116,123,124,151,153,163,196,208,209,221,225-234]
		Subsidies for fruit/veg - Implement subsidies or remove taxes on fruit and vegetables in order to decrease the cost and promote over RPM [227,234]
	Labelling	*Consumer labelling* – eg, package labels that communicate information about the total greenhouse emissions released throughout production and transport of RPM [95,116,122-124,151-153,163,173,193,194,207-209,219,221,233,235,236]
	Availability	*Meat substitution/Alternative proteins* – promotion and increased availability of alternative sources of protein eg, tofu, lentils, cultured meat [116,118,123,237-239]
		Meat ban - government policy that removes availability of RPM available for purchase [123]
		Meat rationing – government policy that sets limits on the amount of RPM available for purchase [123]
	Procurement	*Public food programs* - Set specifications around RPM reduction/replacement for public procurement by government institutions including schools, hospitals, armed forces, and prisons [163,188]
	Advertising and promotion	*Marketing/advertising restrictions* – Restricting marketing of RPM products [32,194]
Consumer Behaviour	Information provision	*Consumer education* – developing public education initiatives or social marketing campaigns that inform consumers on the health/environmental impacts of RPM [23,32,105,117,120,123-126,162,192,219,221,233,238]
	Non-information provision	*Consumer nudging* – eg, incorporating plant semi-manufactures in meat products, in an effort to make eating sustainable products gradually become more accessible [120,238,240]
	Community-led initiatives	*Consumer alliances* – promotion of social movements or advocacy groups aimed at informing consumers and advocating for policy around RPM reduction [238]
