# Supplementary material for: Understanding the Political Challenge of Red and Processed Meat Reduction for Healthy and Sustainable Food Systems: A Narrative Review of the Literature
Source: Int J Health Policy Manag. 2020 Dec 2;10(12):793–808. doi: 10.34172/ijhpm.2020.238 (PMC9309962; doi:10.34172/ijhpm.2020.238)
Supplement: Supplementary file 2 — contains the detailed explanation of the search process. 41-43,70,241,242 [file ijhpm-10-793-s002.pdf]

Supplementary file 2

Methods

This study used a narrative literature review and synthesis method.[41] A narrative literature review is a scholarly summary of evidence that simultaneously incorporates author interpretation and critical analysis.[42] It is appropriate for this study question as it allows for the exploration of relationships within and between studies, as well as thematic analysis of key findings. This method was selected as it allows for both amalgamation and analysis of a broad range of studies that fall under a variety of disciplines and formats. This was deemed important as the topic has been investigated by a wide range of academic disciplines and organisations. To reduce the inherent subjectivity of the narrative review method and to strengthen rigour, a systematic search was performed, guided by the PRISMA guidelines.[43] The method involved four steps: (1) exposition of theory to guide both the search and the analysis; (2) a systematic search for relevant literature, with additional branching searches where necessary; (3) analysis of the literature sources and identification of key themes, and (4) thematic synthesis of the results. One objective and related questions will be addressed in this review:

1) **Understand political economy barriers and enablers to policy implementation:** What is known about political economy factors as barriers and enablers for achieving RPM reduction, including the power of key interest groups?

Search Strategy

A scoping review was performed initially to both identify and group relevant search terms, as well as to identify an appropriate guiding framework. In addition, consultation with a Deakin University Research Liaison Librarian was undertaken in order to ensure full comprehensiveness of search terms, as well as reviewing effective search strings and suitable databases for searches. Using key search terms and following PRISMA guidelines,[43] four databases (Scopus, Web of Science, PubMed, and EBSCO Host) were searched for relevant studies. As is consistent with the narrative literature review method, additional branching searches were conducted iteratively in order to ascertain comprehensiveness of results. Organisational websites were also searched for any relevant reports, policy briefs, or general documents. Organisations included were authoritative bodies with a mandate or interest in improving nutrition, public health and/or environmental

sustainability, including WHO, FAO, Committee on World Food Security, IPCC, Food Climate Research Network (FCRN), International Panel of Experts on Sustainable Food Systems (IPES-Food), EAT, United Nations Standing Committee on Nutrition (UNSCN), International Fund for Agricultural Development (IFAD), World Bank, and World Economic Forum (WEF). A detailed search diary was used throughout the search process (Supplementary file 1) in order to document any adjustments to the search strategy, as well as recording the yield from each database and removal of duplicates.

Search Terms

Terms searched were centred on three key concepts: RPM consumption/production, political economy, and health and sustainability. Various combinations of search terms included "red meat" OR "processed meat" OR "beef" OR "plant based diet" AND "govern*" OR "enabling environment" OR "power" OR "commercial determinants" AND "health" OR "nutrition" OR "sustainab*" OR "planet* bound*". The complete search is detailed in Table 1. A date range of 2000-2019 was set as the scoping review revealed a surge in publications from 2000 onwards. 'Related articles' as suggested by electronic databases were followed up to ensure that key studies were captured in the search.

Table S2. Search Terms and Data Sources Used in Review

Data sources	Terms
PubMed, Scopus, Web of Science, EBSCO Host (Global Health, Medline, Health Policy, EconLit, Political Science),<br><br>Google search, advanced Google search of websites: WHO, FAO, Committee on World Food Security, IPCC, FCRN, IPES-Food,	**Concept 1:** "meat" OR "animal source*" OR "red meat" OR "processed meat" OR "beef" OR "plant based diet" OR "low carbon diet"<br>**Concept 2:** "polic*" OR "politic*" OR "govern*" OR "enabling environment" OR "power" OR "commercial determinants" OR "framework" OR "solution" OR "stakeholder*" OR "politic* econ*" OR "industry"<br>**Concept 3:** "health" OR "nutrition" OR "sustainab*" OR "planet* bound*" OR "climate change" OR "climate change mitigation"

EAT, UNSCN, IFAD, World Bank, WEF	**NOT:** "Veterinary" OR "Energy" OR "police" OR "policing"

Inclusion and Exclusion Criteria

Both qualitative and quantitative academic studies, as well as grey literature were included in this review. Table S3 outlines the inclusion and exclusion criteria. Studies must have been in English, and published after 2000 in order to be included. The year 2000 was selected as the cut-off year as the scoping review revealed an increase in relevant publications from this year onwards. We chose to exclude studies that identified industry measures such as farming technologies as a sole means to improve the impact of agriculture on the environment as this comprised a large number of studies that involved technology change, rather than policy actions.

Table S3. Inclusion and Exclusion Criteria for Review

**Inclusion**	**Exclusion**
Published after 2000 in a peer-reviewed journal or as a substantive report by an authoritative organization (eg, WHO, FAO)	Published prior to 2000.
Had an abstract in English	Abstract not in English.
Full article accessible	Editorials, commentaries and similar
Identified proposed **policy** actions for attenuating red/processed meat production or consumption, and/or considers **political economy** factors driving or attenuating meat production/consumption.	Identified **clinical intervention** actions for attenuating red/processed meat consumption<br>Identified **technologies** or **industry measures** for attenuating red/processed meat production

Analysis

Data Extraction

Titles and abstracts of all included articles were screened. Where title and abstract lacked sufficient detail, full-text versions were retrieved for review. Furthermore, grey literature was systematically searched using the "site or domain" function in order to refine results. Data extracted from studies included that of; author background, author discipline, focus of study, policy actions (proposed or existing), and/or any political economy barriers or enablers identified.

Coding and Analysis

All included articles (both academic and grey literature) were uploaded to and coded using the qualitative analysis software package NVivo12.[70] An initial coding schema was taken from the guiding political economy framework (as illustrated above), which was then iteratively modified and added to throughout the coding process. Consistent with a thematic synthesis approach, the coded data was refined into a final set of themes, and then summarised. Any outlier codes that did not fit the scheme were excluded from analysis unless deemed important during critical evaluation.[241,242] The final set of classifications were assessed in order to determine key findings.
